# OpCitance: Citation contexts identified from the PubMed Central open access articles

**DOI:** 10.1038/s41597-023-02134-x

**Published:** 2023-04-28

**Authors:** Tzu-Kun Hsiao, Vetle I. Torvik

**Affiliations:** School of Information Sciences, University of Illinois at Urbana-Champaign, 501 E. Daniel Street, Champaign, IL 61820 USA

**Keywords:** Research data, Publication characteristics

## Abstract

OpCitance contains all the sentences from 2 million PubMed Central open-access (PMCOA) articles, with 137 million inline citations annotated (i.e., the “citation contexts”). Parsing out the references and citation contexts from the PMCOA XML files was non-trivial due to the diversity of referencing style. Only 0.5% citation contexts remain unidentified due to technical or human issues, e.g., references unmentioned by the authors in the text or improper XML nesting, which is more common among older articles (pre-2000). PubMed IDs (PMIDs) linked to inline citations in the XML files compared to citations harvested using the NCBI E-Utilities differed for 70.96% of the articles. Using an in-house citation matcher, called Patci, 6.84% of the referenced PMIDs were supplemented and corrected. OpCitance includes fewer total number of articles than the Semantic Scholar Open Research Corpus, but OpCitance has 160 thousand unique articles, a higher inline citation identification rate, and a more accurate reference mapping to PMIDs. We hope that OpCitance will facilitate citation context studies in particular and benefit text-mining research more broadly.

## Background & Summary

Citing prior work has long been a common practice in academic writing. In general, citations were used by authors to situate the reported work within the scope of the subject field and provide intellectual linkage between past and the reported work. Hence, citations have been broadly used for tracking the advance of science, accessing development of disciplines, and evaluating the impact of research output^1–5^. However, previous studies^6–9^ showed that not all citations were equal, and scholars cited prior work for various kinds of purposes.

Many efforts have been put into studying the reasons for making citations and the importance of cited work to the citing work. Aside from surveying and interviewing authors^10–13^, analyzing citations using full-text articles (i.e., inline citations) provides an unobtrusive way for scholars to explore the motivation and importance behind each citation. Count-based features, location-based features, and textual features were the three popular categories of features used in previous studies. Count-based features measured the count of occurrences of a cited work in the text. For instance, the number of times for a cited work being mentioned in the entire citing article was reported as an informative feature for identifying important citations^14^. Location-based features provided insights about the role played by the cited work in the citing work^8,15–17^. For example, citations found in the introduction and literature review could be cited for providing background knowledge or supporting research arguments, while citations found in the result or discussion section implied comparisons between past and the reported research findings^8,15^. Zhao and Strotmann^18^ explored the influence of filtering out citations in introductory and background sections on evaluating authors’ research impact. On the other hand, textual features used semantic cues extracted from the text surrounding citations to capture authors’ motives for making citations^9,19–21^. These surrounding texts are known as citation contexts. The window of a citation context can be a fixed number of characters, the citing sentence (sometimes including its preceding and following sentences), or a text block containing sentences related to the cited article^22–25^. In this study, the window for a citation context is defined as a sentence where a citation appears (i.e., the citing sentence).

Although studies on inline citations have been developed for more than forty years^26^, theories and methods of capturing the motivations behind citations and measuring the importance of cited work to the citing work are still in progress. One of the challenges is acquiring data. Citation studies used to rely heavily on bibliographic data obtained from bibliography databases such as Web of Science (WoS) and Scopus. These databases have limited access to full-text articles and require subscriptions. The movement to make scholarly articles open access (OA) has gradually changed the landscape. Piwowar *et al*.^27^ used DOIs to estimate the percentage of scholarly articles that were open access and found that it ranged from 27.9% to 47.0%, depending on the source of the DOIs and the time of publication. In line with the OA trend, citation data is no longer restricted to subscription databases^28^. For instance, Crossref provides APIs for retrieving citation links. The NIH Open Citation Collection (NIH-OCC)^29^ provides open citation data for PubMed articles. Although the open science trend and the massive growth of OA articles allow for large-scale studies of inline citations and citation contexts^30–32^, it is still not an easy task to identify inline citations from full-text articles. Efforts have been made in the computational linguistics and computer science communities. The ACL anthology network (AAN) corpus^33^, the Semantic Scholar Open Research Corpus (S2ORC)^34^, the COVID-19 Open Research Dataset (CORD-19)^35^, and a dataset collected using the Academic Citation Typing (ACT) platform^36,37^ are the existing large-scale citation contexts datasets. The AAN corpus contains 77,753 citation contexts from 18,290 articles, and S2ORC contains over 12 million full-text articles with inline citations annotated^33,34^. The ACT dataset contains 11,233 citing sentences annotated by six citation purposes (*background, uses, compare_contrast, motivation, extension*, and *future*)^36,37^. Part of the ACT dataset has been enhanced with 12 features and released as the ACT2 dataset^38^. These datasets were generated from PDF version of articles. CORD-19 contains over 72 thousand full-text articles on COVID-19 and related historical coronaviruses^35^. These articles were sourced from PDF and XML versions of articles: The PDF version of articles were from PubMed, PubMed Central (PMC), the World Health Organization’s COVID-19 database, and preprint servers (bioRxiv, medRxiv, and arXiv); the XML version of articles were from PMC^35^. Here, we introduce OpCitance, a dataset generated from the XML version of the articles in the PMC Open Access Subset (PMCOA subset) (https://www.ncbi.nlm.nih.gov/pmc/tools/openftlist/). OpCitance contains all sentences from over 2 million articles. For sentences with inline citations, the inline citations and their PMIDs (if applicable) are annotated. These sentences are defined as citation contexts (or citances) in OpCitance. The sentence-level information retrieval and extraction focus have a long history in PubMed and PMC, such as the National Library of Medicine’s (NLM) LitSense^39^ search system and SemRep^40^ information extraction system. The OpCitance data is complementary to these tools and can be combined in new types of text-mining and citation analysis.

Journal Article Tag Suite (JATS) is a standardized markup scheme for tagging journal articles in the XML format (https://jats.nlm.nih.gov/about.html). JATS was developed by the National Library of Medicine (NLM) and currently maintained by the National Information Standards Organization (NISO). In PMC, JATS was adopted as the preferred XML tagging style for article submissions. The unified XML tagging style gave the possibility to automatically extract citation contexts at a large-scale. In this study, we developed an XML parser which could process XML files in the PMCOA subset and meet the following goals: (1) parsing each article into sentences, (2) identifying citation contexts and PMIDs of the cited work, (3) identifying section titles and mapping section titles with standardized IMRaD structure (introduction, method, results, and conclusion and discussion), and (4) labeling each sentence by text progression and the corresponding IMRaD category.

This article describes the method of identifying inline citations and their citation contexts from the PMCOA subset and makes OpCitance available to the public. To construct this dataset, we collected the PMCOA subset in May 2019. At the time of data collection, there were 2,407,660 articles in the PMCOA subset, in which 2,049,871 articles had at least one identifiable citation context. This dataset could benefit scientists interested in studying citation motives and citation behaviours. Moreover, this dataset could be used for text-mining projects such as studying scientific writing styles and other citation analysis research.

## Methods

This section describes how inline citations and citation contexts were extracted. In OpCitance, an inline citation refers to a citation that appears in a paragraph, table, or figure/table caption, whereas a citation context refers to the sentence or table cell that contains the inline citation. Below is an example of a citation context produced by our XML parser for PMCID: 5219817 with two inline citations denoted by |B1| and |B2|.


If for any reason this process fails, gradually the person will suffer from osteoporosis |B1|, |B2|.


**Fig. 1:**
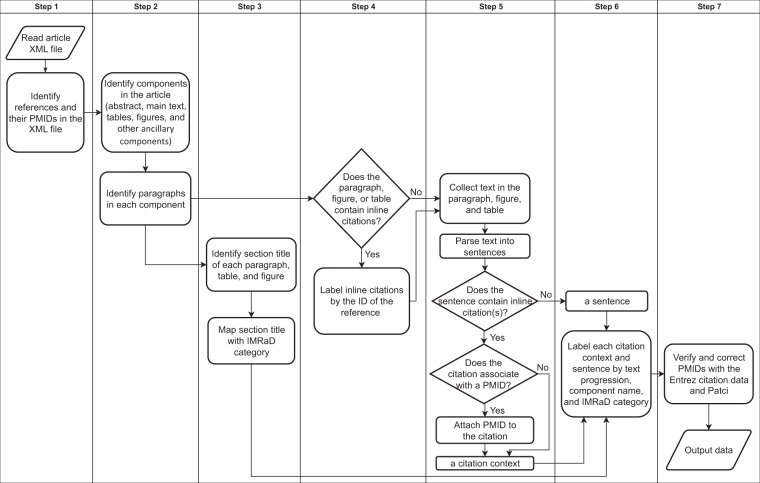
Workflow of generating the dataset.

XPath syntax was used to navigate through the XML tags. Python’s lxml package was utilized to parse XML files because it had better compatibility with XPath syntax. Figure 1 depicts the overall workflow of generating the dataset. First, references and their PMIDs (if any) were identified. Second, the components (abstract, main text, tables, figures, and other ancillary components) in each article and the paragraphs in each component were identified. Third, the section titles of paragraphs, tables, and figures were extracted and mapped to the IMRaD categories. Fourth, inline citations in each component were marked. Fifth, the text was parsed into sentences and the citation contexts were identified. For each inline citation per citation context, the PMID was appended if the XML file had a PMID for the inline citation. Sixth, citation contexts and sentences were labeled by text progression, component names, and the belonging IMRaD categories. Seventh, the PMIDs were verified with citation data collected from the NCBI Entrez Programming Utilities (https://www.ncbi.nlm.nih.gov/pmc/tools/cites-citedby/) (hereinafter referred to as the Entrez citation data) and Patci^41^, a citation matcher. Specifically, the NCBI Entrez Programming Utilities take PMIDs of the PMC articles as inputs and return lists of PMIDs cited by each input PMID. Patci matches reference strings to records from a set of bibliographic databases (e.g., PubMed, DBLP, and ADS) and returns the source link IDs (e.g., PMIDs) and the match probability of each ID. The details of each step are addressed in the following sections.

### Identifying references and their PMIDs from XML files

**Fig. 2:**
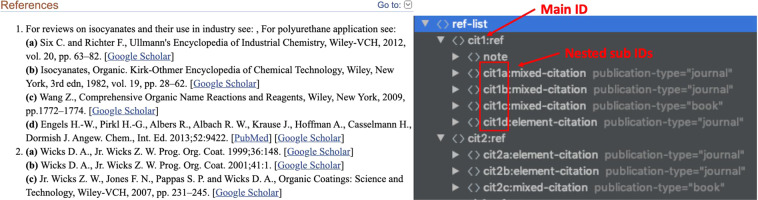
Example of nested references. (left: text shown in article; right: XML structure).

References were identified through the <ref> tags. Typically, one <ref> tag pointed to one reference, and the ID of the reference (referred to as ref-ID below) could be identified through the “id” attribute of the <ref> tags. However, in the cases that multiple references nested under one <ref> tag (as shown in Fig. 2), the IDs of the nested references were collected through the “id” attributes with tag names containing *citation* (e.g., <mixed-citation>, <element-citation>, and <nlm-citation>). Aside from collecting the IDs given by the publisher, we also searched whether references had PMIDs. The PMIDs were identified through the “pmid” attribute associated with <pub-id> tags.

### Identifying components in an article

A set of tags were used to identify abstract, main text, tables, figures, and other ancillary components (e.g., glossary, appendix, and conflict of interests). Abstracts were identified through <front//abstract> and <front//trans-abstract> tags. Main text was identified through the <body> tag. Tables and figures were retrieved through tag names starting with <table-warp> and, or through the <tbody> tag under <array> tag. Ancillary components were identified through the <back> tag. After the components were identified, <p> and <disp-quote> tags were used to find text fragments in each component.

### Mapping section titles to IMRaD categories

The IMRaD categories were identified using section titles and section type information extracted from the XML files. We decided to use IMRaD categories as standardized section types for two reasons: First, the IMRaD structure has been widely adopted in the scientific literature since the 1970s^42^. Second, empirical studies on inline citations have utilized the IMRaD categories for analyzing citation functions, sentiments, and importance^43,44^. Providing the IMRaD categories could be beneficial to future research on similar topics. The section titles were extracted through the <title> tags or <label> tags being the child node of <sec> tags, and the section type information was extracted through the “sec-type” attribute. The section titles and section types were then concatenated into a string and processed by a rule-based matching algorithm. The algorithm matched the string with a set of cue words and phrases (Table 1). If a match was found, the corresponding IMRaD category would be returned. To identify the cue words and phrases, we sorted section titles and section types by the number of occurrences. Section titles and types with high occurrences were manually inspected, and the cue words and phrases commonly used in scientific articles for denoting the IMRaD categories were identified. We understood that this rudimentary approach might cause some misidentifications, and the IMRaD categories of some sections (e.g., introduction, background, and discussions) could be easier to identify than the other sections. However, the distribution of the identified IMRaD categories along with text progression (see Technical Validation below) was aligned with the common structure of scientific articles.

**Table 1:** Terms for mapping section titles with standardized section types.

Label	IMRaD category	Cue words and phrases
I	Introduction/Background	intro*, overview, background, history, related work, related stud*, previous work, previous stud*, review
M	Method	method, material, experimental procedure, protocol, data
R	Result	result, finding
D	Conclusion/Discussion	conclud*, conclusion, summary, discuss*, future
NoIMRaD	—	The string does not contain the above terms.

### Finding inline citations

Inline citations were identified through the <xref> tags. In JATS, <xref> tags represent cross-references to objects within the document. The referred object can be a table, a figure, a citation, etc. To verify whether a <xref> tag was indeed pointing to a citation, we collect the ID of each <xref> tag through the “rid” attribute (addressed as xref-ID below). Each of the xref-IDs was then matched with the ref-IDs. Only the xref-IDs matched with ref-IDs were marked as inline citations.

It is worth noticing that citations could be implicitly mentioned in text in some referencing styles. The common cue of an implicit mention was a hyphen between the citation markers such as “[3–6]” or “(3–6)”. In cases like this, not every inline citation was tagged in full-text articles but could be inferred from the citation markers. These untagged inline citations were the implicitly-mentioned citations.

Two tagging styles of implicitly-mentioned citations were found in the XML files. The first one was wrapping the implicitly-mentioned citations by two <xref> tags; the second one was using one <xref> tag to represent a set of citations. Examples of the two tagging styles would look like “[3–6]” and “[3–6]”, respectively. The underlines in the examples denoted the citation markers associated with <xref> tags. For the first case, the implicitly-mentioned citations were identified through enumerating the citations between the two <xref> tags. For the second case, the enumeration went until the end of the label (e.g., the “6” in [3–6]).

### Identifying citation contexts and sentence labelling

Along with the process of searching inline citations, the text in the paragraphs, tables, and figures was also collected. Also, when a citation was identified, the citation marker was replaced by the ID of the citation, wrapping by two vertical bars (e.g., |ref1|). After the above labelling process, the collected text was parsed into sentences. The Natural Language Toolkit (NTLK library) was used for tokenizing text found in paragraphs and figure captions into sentences while text in tables was collected element by element. NLTK was selected for sentence tokenization (i.e., parsing text in paragraphs and figure captions into sentences) since it is a well-established library for processing biomedical articles^39,45,46^ and because it is fast. For a typical article in our dataset, NLTK takes about 1 second to tokenize all sentences, while the Stanford NLP group’s Stanza library takes about 3 seconds. Sentences containing citations were citation contexts. Furthermore, citation contexts and sentences were labeled by the belonging component names (abstract, body, etc.) and IMRaD categories identified in the above steps. Note that IMRaD identification was not applied to abstracts and ancillary components. The IMRaD labels for sentences in abstracts and ancillary components (e.g., glossary, appendix, and conflict of interests) are all ***NoIMRaD***. The labels for the component names and the IMRaDs could be found in the dataset’s *location* and *IMRaD* columns, respectively. If a citation had a PMID from the XML file, the PMID was also attached.

### Verifying and correcting PMIDs identified from XML files

The cited PMIDs identified from the XML files (hereinafter referred to as the XML-tagged PMIDs) were verified and corrected with two approaches. First, the XML-tagged PMIDs were compared to the Entrez citation data (as of December 2018), which included citations from 4,243,594 PMC articles to PubMed articles. The *intxt_pmid_source* indicator was created as a result of this comparison: if the XML-tagged PMID could be confirmed by the Entrez citation data, it received the value *xml,pmc*; otherwise, it received the value *xml*. Second, we determined the best source link IDs for the references (e.g., PMIDs and non-PMIDs: DBLP IDs and ADS IDs) and mapped these IDs to the inline citations. The best IDs for 98.25% (135,340,795) of the OpCitance’s 137,748,787 inline citations were identified using Patci, a tool that took reference strings (extracted from the XML files) as input and returned source link IDs as well as the match probability of each ID. The best ID of each reference string was determined with a match probability threshold. The default threshold is 0.997 and was lower for non-PMIDs or for PMIDs that could be confirmed by one of the nine public sources such as iCite, the Entrez citation data, and OpenCitations. These sources are listed by name in a field in OpCitance. The harvested and cleaned citation data for PubMed articles from the nine public sources is derived from an inhouse project tentatively called uCite for which the working manuscript is available by request. The thresholds were set by manually inspecting a collection of references that appeared to be borderline. Furthermore, PMID was the preferred ID unless the non-PMID’s match probability was considerably higher. The best IDs for the remaining 1.75% (2,407,992) of inline citations were the XML-tagged PMIDs (if any). These 2,407,992 inline citations that were not cross-checked with Patci were mainly due to the citing papers’ publication years. Patci is based on a snapshot of PubMed citation data collected in 2018. Out of these 2,407,992 inline citations, 2,284,590 (94.88%) are from papers published in 2018 or later. Each best ID has two indicators: The *best_source* indicator states the sources that confirm the ID (e.g., *xml,pmc,mag*); the *best_id_diff* indicator (Table 2) denotes the comparison result between the XML-tagged PMID and the best ID.

**Table 2:** Definition of the *best_id_diff* indicator values.

*Best_id_diff* Indicator value	*Best_id* value	Definition	*Best_id* of the citation is cross-checked with Patci
SAME	Patci-identified ID	The Patci-identified ID is the same as the XML-tagged PMID.	Yes
NONE	—	The citation has neither a Patci-identified ID nor an XML-tagged PMID.	Yes
INSERT	Patci-identified ID	The citation has a Patci-identified ID but lacks an XML-tagged PMID.	Yes
SWAP	Patci-identified ID	The Patci-identified PMID is different from the XML-tagged PMID.	Yes
DELETE	—	The citation has an XML-tagged PMID, but Patci does not identify any ID for it.	Yes
PMID_XML	XML-tagged PMID	The citation has an XML-tagged PMID.	No
NONE_XML	—	The citation does not have an XML-tagged PMID.	No

Further details of the comparison results are addressed in the Technical Validation section.

## Data Records

The May 2019 XML version of the PMC open access subset contains 2,407,660 articles, of which about 85.14% of articles (2,049,871 articles) have a reference section and at least one <xref> tag pointing to a reference (i.e., having at least one inline citation). These 2,049,871 articles contain 720,649,608 sentences (a text cell in a table counts as a sentence). Of these 720,649,608 sentences/text cells, 75,848,689 (10.53%) are citation contexts, yielding 137,748,787 inline citations. On average, there are 1.82 inline citations per citation context. These 137,748,787 inline citations include 86,035,875 references that account for 99.49% of the total of 86,473,346 references (0.51% of references do not have citation contexts). As stated in the Method section, not all inline citations were tagged with the <xref> tag. Of the 137,748,787 inline citations, 127,810,293 (92.79%) were captured through <xref> tags, and 9,938,494 (7.21%) were implicitly-mentioned citations (extracted from citation markers associated with a <xref> tag but pointing to multiple references, e.g., [3–6]) identified by our XML parser.

**Fig. 3:**
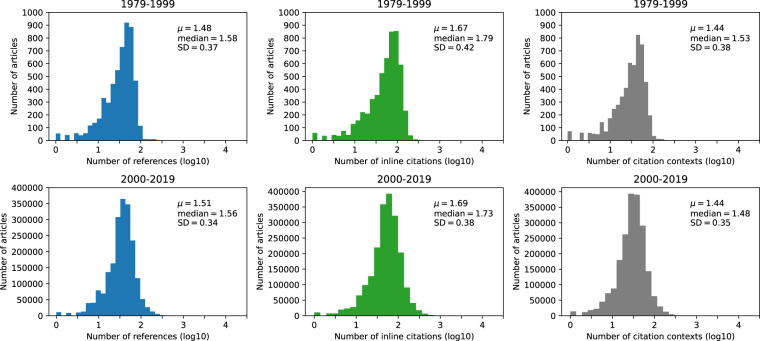
Distribution of numbers of references and inline citations. For articles published between 1979 and 1999, the mean number of references, inline citations, and citation contexts were 30.30, 47.04, and 27.80, respectively. As for articles published between 2000–2019, the mean number of references, inline citations, and citation contexts were 32.47, 48.64, and 27.82, respectively. These numbers were the antilogarithms of the means presented in the figure.

The 2,049,871 articles having at least one inline citation and its citation context identified were published in 8,770 journals. The years of publication spread from 1979 to 2019. Although PMC was launched in early 2000, PMC had digitalized back issues of historically-significant biomedical journals up to 1923 for US journals and up to 1877 for foreign journals. In OpCitance, 5,449 (0.27%) articles were published prior to 2000, and 2,044,422 (99.73%) were published after 2000. Figure 3 presents the distribution of the number of references, the number of inline citations, and the number of citation contexts in the two periods (1979–1999 and 2000–2019). On average, the number of references and the number of inline citations increased in the later period. The mean number of references increased from 30.30 to 32.47, and the mean number of inline citations increased from 47.04 to 48.64. For articles published in 2000 and after, the number of references, inline citations, and citation contexts on log scale appear to follow a normal distribution, except for an excess portion in the left tail that are likely due to shorter types of articles (e.g., letters). 95% of the articles have between 5–122 references, 6–214 inline citations, and 4–115 citation contexts. It is also worth noticing that publication types are more diverse in the second period. Articles published before 2000 only covered 11 publication types, and the most common type, *research article*, accounted for 90.95% of the articles, while 1.96% were *review articles*. In 2000 and after, there were 33 publication types, and *research articles* dropped to 78.81%, while *review articles* increased to 7.71%. This helps explain the increase in articles with more than 100 references in the second period.

**Fig. 4:**
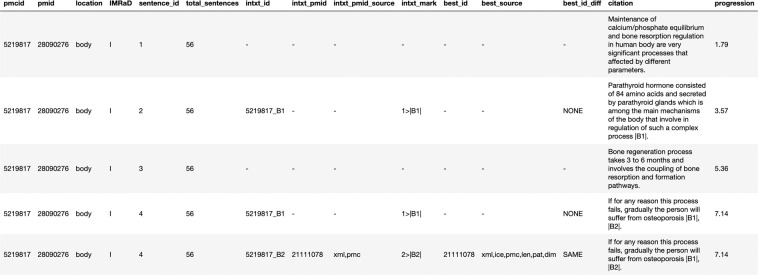
Snapshot of data records in OpCitance.

Figure 4 shows a snippet of data records in OpCitance. The data files are formatted as tab-separated values (TSV). Each row in the dataset contains a citation context or a sentence associated with fourteen attributes. The columns, *pmcid* and *pmid*, are the unique identifiers of the citing article in PMC and PubMed, respectively. *Location* gives information about article component (abstract, main text, table, figure, etc.) where each citation context/sentence belongs. *IMRaD* addresses the IMRaD section where each citation context/sentence is in. *Sentence_id* provides the ID of the citation context/sentence in the component. Notably, when a citation context contains more than one inline citation, the citation context appears as multiple rows in our dataset, but the *sentence_id* of the citation context remains the same. *Total_sentences* is the number of sentences in the component. *Intxt_id* records the unique identifier of the cited work. *Intxt_pmid* records the PMID of the cited work (if any) retrieved from the XML files (i.e., the XML-tagged PMID). *Intxt_pmid_source* addresses the source where PMIDs were identified: *Xml* represents that a PMID is only identified from the XML file, while *xml,pmc* represents that the PMID is not only from the XML file, but also in the Entrez citation data. *Intxt_mark* provides the citation marker associated with the inline citation. *Best_id* records the best source link ID (e.g., PMID) for each inline citation. *Best_source* addresses the sources that confirms the *best_id*. *Best_id_diff* indicates the comparison results between the *best_id* and the *intxt_pmid* columns. *Progression* records text progression of each citation context/sentence.

OpCitance has been deposited to the Illinois Data Bank^47^:

10.13012/B2IDB-4353270_V2

The dataset contains 24 TSV files. The first 15 files are the articles published in journals with journal titles starting from A to O. The 16^th^ and 17^th^ files are the articles published in journals with journal titles starting with P. The 18^th^ to 23^rd^ files are the articles published in journals with journal titles starting from Q to W. The last file contains the articles published by journals with journal titles starting with X, Y, or Z.

## Technical Validation

As addressed in the Data Records section, 99.49% of the references’ inline citations were identified. Although only 0.51% of the references were without inline citation, this condition might still affect the future use of the dataset. Hence, in the following sections, we addressed the distribution of references without inline citation by the following characteristics of citing articles: publication years, publication types, and the sources of the XML files. Also, a probabilistic model was used to assess the effect of the above features on the likelihood of an inline citation of a reference being identified.

### Publication year and publication type

**Fig. 5:**
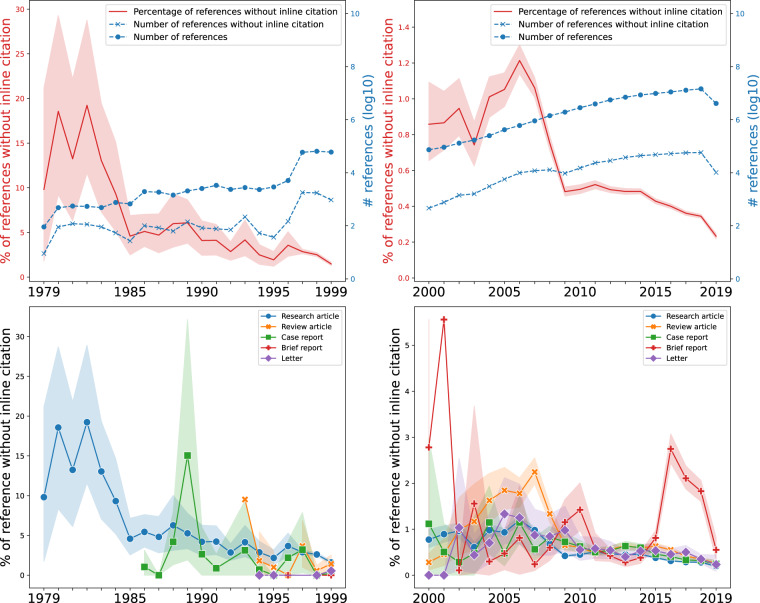
Distribution of references without inline citations by publication years (upper-left and upper-right) and publication types (bottom-left and bottom-right). The shaded area showed the percentage of references without inline citations with 95% confidence interval.

Figure 5 showed how the inline citations of references were identified in different publication years and publication types. Overall, the percentages of references without inline citations dropped as time progressed, implying the tagging of the XML files improved over time. These improvements followed distinct patterns that appeared in the four following periods: 1979–1984, 1985–1999, 2000–2008, and 2009–2019. In the first time period, missing inline citations were frequent (between 7%–20% of the references), but this time period contains a small portion of the entire dataset (0.003%, 2,912 references). In the second period, the proportions of references without citation contexts dropped gradually from around 5% to around 1%. The proportion of references without citation contexts remained around 1% in the third period, and dropped from 0.4% to 0.2% in the fourth period. The patterns observed before and after the launching year (2000) of PMC implied that although PMC digitalized back issues of journals, some of the citations might not be captured and tagged in the digitalization process.

The bottom panels in Fig. 5 showed the percentages of references without inline citations in the five most common publication types (research article, review article, case report, brief report, and letter). These five publication types accounted for 94.97% (1,946,687) of the articles in OpCitance. Each of the five categories followed a pattern similar to the overall trend, but with some notable exceptions. In review articles, inline citations were missing at almost twice the rate of other types of articles in 2004–2008. In brief reports, the rates were nearly five times as the other types of articles between 2016 and 2018.

### Sources of XML files

**Fig. 6:**
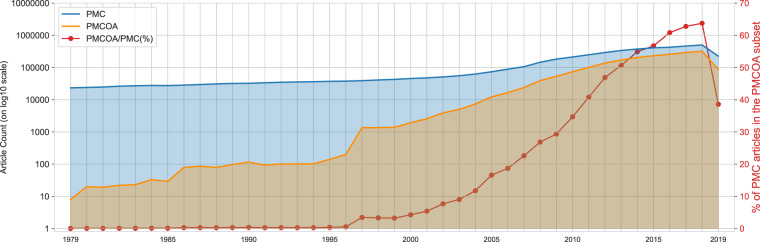
Number of articles in the PMC and the PMCOA subset by publication years. The blue line and the orange line show the number of articles in the PMC and in the PMCOA, respectively. The red line shows the percentages of PMC articles that are in the PMCOA subset.

Articles are deposited into PMC by participating journals and authors e.g., who are required to make their articles publicly accessible due to NIH funding. Participating journals deposit XML files following JATS (see Methods). Author manuscripts (e.g., Word, PDF) are processed through the NIH Manuscript Submission (NIHMS) system and converted to JATS. Moreover, there were three kinds of participating journals: full participation, selective deposit, and NIH portfolio. While a journal is in full participation journals, they deposit all their articles. Articles from selective deposit journals are mainly due to authors opted to pay for open-access. Articles in the NIH portfolio are the articles where authors acknowledge NIH funding. Note that the PMC open-access subset consists of PMC articles under Creative Commons (CC) or similar licenses. In other words, the articles in PMC subject to traditional copyrights restrictions are not in the open-access subset, although they are free to access individually as PDF files. Also, some journals delay the release of articles in PMC. The delays were mostly within a year, but can be more than one year for some journals (e.g., *Journal of the Royal Society of Medicine*, 36-months delay). To estimate the proportion of the open-access subset in the PMC, we searched PMC for articles published between 1979 and May 2019. (The query is *“YYYY/01/01”[Publication Date]:“YYYY/12/31”[Publication Date]* for each year from 1979 to 2018. For year 2019, the query is *“2019/01/01”[Publication Date]:“2019/05/31”[Publication Date]*.) Overall, open access articles accounted for about 41% of the articles indexed in PMC, and the proportion of open access articles in PMC increased over time (Fig. 6). Very few old PMC articles are open access (less than 1% between 1979–1996). From 1997 to 2018, the shares of open access articles in PMC increase from 3% to 64%. Note that PMCOA statistics are from the XML files downloaded from PMC in May 2019, and the PMC statistics were collected during preparation of the manuscript (September 2022). The drop in 2019 is likely to be an artifact due to the delay deposit policies of journals, and the time gap between PMC indexing and deposit in the PMC’s FTP bulk download.

To acquire the sources of XML files, we download the PMC journal list (https://www.ncbi.nlm.nih.gov/pmc/journals/). The journal list covers full participation and NIH portfolio journals. Journal titles not on the list are selective deposit journals. PMCIDs of author manuscripts were retrieved from PMC using the query, *author manuscript[filter]*. Journal titles and PMCIDs of the XML files of the PMC open access articles were then mapped with the journal list and PMCIDs of author manuscripts for labeling each file’s deposit source. Note that this assigns a fixed participation property to each journal, but it is possible for journals to vary over time. For example, full participation and NIH portfolio journals could move to selective deposit model at some point. In the PMC journal list, PMC marked these journals as “Now Select” and denoted the most recent issues under full participation/NIH portfolio. For these journals, articles with publication years greater than the year of the corresponding most recent issue were labeled as *selective deposit* in the mapping process. Table 3 showed the percentages of references without inline citations in articles from the deposit sources. Selective deposit journals had the highest proportion of references without inline citations, and full participation journals had the lowest proportion of references without inline citations.

**Table 3:** Percentages of references without inline citations in different deposit sources.

Deposit Source	# Articles	# References	# References without inline citations (%)	
Full participation	1,830,722	76,275,089	325,347	(0.43)
Selective deposit	138,356	6,763,865	87,587	(1.29)
NIH portfolio	58,357	2,395,980	17,018	(0.71)
Author Manuscript	22,436	1,038,412	7,519	(0.72)
Total	2,049,871	86,473,346	437,471	(0.51)

**Fig. 7:**
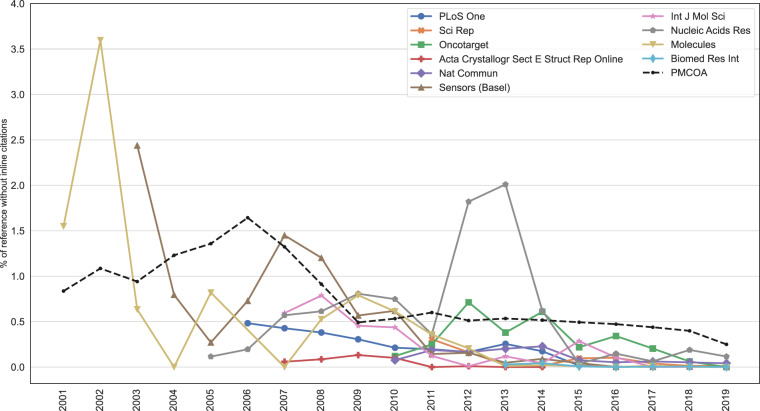
Percentages of references without inline citations in ten journals with highest publication counts.

Since journals deposited the great majority of articles (98.91%), it was likely that the publishers/journals had some roles in the tagging quality of the inline citations. To access this, Fig. 7 showed missing inline citations in the ten largest journals in the dataset. Note that large journals such as *Science, Nature, PNAS*, and *BMJ* were not in the ten journals because most of articles published by these journals were not CC-licensed. For instance, *PNAS* had 120,232 articles indexed in PMC (from 1979 to May 31, 2019) but only 1,840 were in our dataset.

The results implied that large journals might have better tagging quality. After 2010, the missing rates were below 0.5% and were lower than the full dataset with a few exceptions. In 2012 and 2014, the missing rates of *Oncotarget* were slightly higher than the full dataset. As for *Nucleic Acids Research*, two peaks were observed in 2012 and 2013, with the missing rates almost four times as the full dataset’s missing rate. Also, for six journals (*PLOS ONE, Scientific Reports, Acta Crystallographica Section E: Structure Reports Online, Nature Communication, International Journal of Molecular Sciences*, and *BioMed Research International*) having missing rates lower than the full dataset across all publication years; five out of six (except for *BioMed Research International*) were born as electronic journals.

**Fig. 8:**
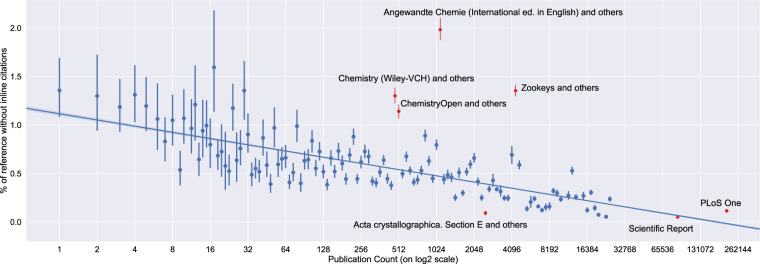
Relationship between journal size (in PMCOA subset) and missing inline citations. The bars show the 95% confidence intervals. The regression line is based on data that excludes outlier journals as labeled in red.

Figure 8 shows the relation between journal publication counts and the missing rates. In general, large journals have lower missing rates, but there are some exceptions. *Chemistry (Wiley-VCH)*, *ChemistryOpen*, *Angewandte Chemie (International ed. in English)*, and *Zookeys* had high missing rates. To explore the possible reason for the high missing rates, we randomly sampled and manually inspected ten articles from each journal. For the first three journals, the missing was mostly because the reference list was not structured as JATS’ recommendation. According to JATS, when multiple works are placed into a reference (e.g., references 1a, 1b, and 1c in reference 1), each work (i.e., 1a, 1b,and 1c) should be tagged by either <element-citation> or <mixed-citation> tag and nested under a <ref> tag. However, in these three journals, our manual inspections found that the XML files treated nested works as separate references. In other words, reference 1, 1a, 1b, and 1c were tagged by four different <ref> tags where reference 1 was empty. This situation would be problematic when a citation context pointed to reference 1 for citing 1a, 1b,and 1c together. In cases like this, since the nested works were not tagged under a <ref> tag, our algorithm could not capture these works due to lacking the nesting structure and captured an empty reference instead. For the journal, *Zookeys*, we found that the <xref> tags of some references were missing (i.e., the citation markers were plain strings without <xref> tags), and a few references did not appear in the full text.

### A probabilistic model of context identifiability

Overall, 0.5% of references lack inline citations, while 99.5% of references point to one or more inline citations. However, this identifiability rate varies systematically with certain aspects of articles. For example, the most recent year (2019) has the identifiability at 98.8%. In order to understand some of these influences, we built a logistic regression model of identifiability. The model measures the influence of certain aspects on the probability of inline citation identification, as follows:

$Pr(identified\_inline\_citation)=\frac{1}{1+{e}^{-{\beta }_{0}-{\beta }_{1}{x}_{1}-{\beta }_{2}{x}_{2}\ldots -{\beta }_{n}{x}_{n}}}$ where *x*_1_, *x*_2_, …, *x*_*n*_ are the explanatory features. The features include aspects of the citing article, including publication year, publication type, deposit source, and publication venue. More specifically, publication year features include four different phases observed in Fig. 5. The publication venue features include journal size (publication count in PMCOA subset), whether or not it was born as a digital journal, as well as indicators for seven specific journals (two particularly large ones, and five with unusual missing rates as shown in Fig. 8).

The coefficients in Table 4 show how each feature influences the probability of identifying inline citation of a reference. Overall, the identifiability increases over time, but different patterns are shown in the four time periods. Compared with the last period (2009–2019), the probability increases faster in the first period (1979–1984) and slower in the second period (1985–1999) and the third period (2000–2008). The probability of identifying inline citations in *research articles* is higher than the other publication types (*case reports*, *review articles*, etc.). Relative to *full participation* journals, the probability is higher in *selective deposit* journals and *author manuscripts*. However, this is also relative to journal size and the specific journals. For example, *PLOS ONE* and *Scientific Report* are full participation and the two largest journals in our dataset. The identifiability increases with the journal size and is higher in born digital journals. Note that journal size here refers to the number of articles in the PMCOA subset. Some big journals such as PNAS only have a small portion of articles that are open access; hence, the true sizes of these journals are not reflected in our model.

**Table 4:** Logistic regression results.

Feature	Coef.	SE
Intercept	1.281***	0.024
**Publication year (PY)**		
PY-1979^a^	0.064***	0.001
1979–1984	−1.897***	0.107
1985–1999	−0.451***	0.077
2000–2008	0.695***	0.066
(PY-1979) × (1979–1984)	0.110***	0.033
(PY-1979) × (1985–1999)	−0.023***	0.004
(PY-1979) × (2000–2008)	−0.046***	0.002
**Publication type (v.s. Research article)**		
Review article	−0.134***	0.004
Case report	−0.215***	0.011
Brief report	−0.572***	0.010
Letter	−0.388***	0.022
Other	−0.931***	0.007
**Deposit source (v.s. Full participation)**		
Selective deposit	0.376***	0.006
NIH portfolio	−0.090***	0.008
Author manuscript	0.050***	0.012
**Publication venue**		
log_2_(Journal size)	0.171***	0.001
Born as digital journal	0.436***	0.004
PLOS ONE	−0.223***	0.011
Scientific Report	0.619***	0.022
Angewandte Chemie International Edition	−5.017***	0.011
Chemistry (Wiley-VCH)	−4.955***	0.013
ChemistryOpen	−4.778***	0.013
Zookeys	−3.834***	0.008
Acta Crystallographica Section E: Crystallographic Communications	15.065	179.429

*** = *p* < 0.001.

^a^Publication year of citing article minus 1979, which is the earliest publication year in the dataset.

### Citations associated with PMIDs

As addressed in the Methods section, the Entrez citation data contained citations from 4,243,594 PMC articles to articles in PubMed. Within the 4,243,594 PMC articles, 1,818,893 articles were in the PMC open access subset. By comparing the PMIDs retrieved from the XML files (i.e., the XML-tagged PMIDs) and the PMIDs listed in the Entrez citation data, we found that 70.96% (1,290,693 out of 1,818,893) of the articles had at least one discrepancy between the PMIDs of citations. The discrepancies indicated that PMIDs listed in the Entrez citation data were absent in the XML files or vice versa. Based on the discrepancies, we discovered 6.59% (5,148,521 out of 78,085,042) of the citations in the 1,818,893 articles that should have PMIDs but were not tagged in the XML files. Notably, this did not indicate that the citation contexts of these citations were not identified, but showed that the PMIDs of these citation contexts were missing in the XML files.

The discrepancies between the XML files and the Entrez citation data motivated us to further investigate the PMIDs. The source link IDs (e.g., PMIDs, ADS IDs, and DBLP IDs) for 98.25% (135,340,795 out of the total 137,748,787) of the inline citations were identified using Patci and cross-checked with the XML-tagged PMIDs. The Patci-identified IDs and the XML-tagged PMIDs agreed on 91.13% (123,337,645 out of 135,340,795) of the inline citations (SAME: 101,885,318 (75.28%) inline citations; NONE: 21,452,327 (15.85%) inline citations). The disagreement was mostly caused by citations with Patci-identified IDs but without XML-tagged PMIDs (INSERT: 11,595,741 (8.57%) inline citations). Only 0.3% of the citations had disagreement between the Patci-identified IDs and the XML-tagged PMIDs (SWAP: 317,513 (0.23%) inline citations; DELETE: 89,896 (0.07%) inline citations). These results suggest that XML files may have high precision but low recall on tagging the PMIDs associated with the citations. Note that not all Patci-identified IDs are PMIDs. Of the 11,913,254 inserted or swapped IDs, 9,261,870 are PMIDs and 2,651,384 are non-PMIDs (e.g., ADS IDs and DBLP IDs). In other words, Patci supplemented or corrected PMIDs for 6.84% of inline citations (9,261,870 out of the 135,340,795 inline citations).

Of the 2,407,992 (1.75%) inline citations that were not cross-checked with Patci, 1,319,962 (0.96%) had XML-tagged PMIDs. Although only 1.91% (25,179 out of 1,319,962) of these PMIDs were verified with the Entrez citation data, the vast majority of these PMIDs are likely to be correct due to the low SWAP and DELETE rates in the full dataset.

### Evaluation on the identified IMRaD categories

**Fig. 9:**
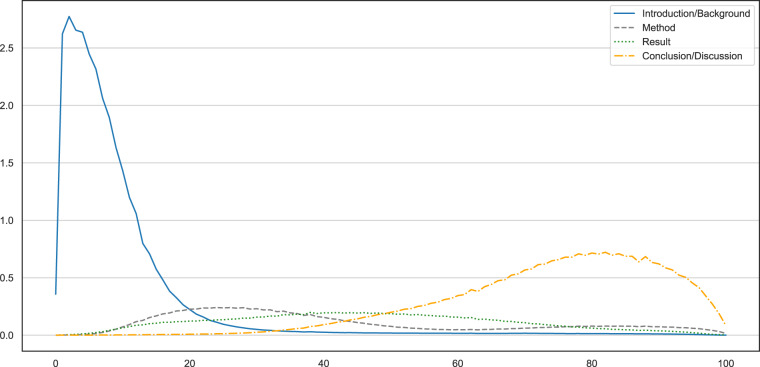
Inline citations in IMRaD sections by text progression. Inline citations concentrated at the beginning and the end of scientific articles. In specific, 28.84% of the inline citations were found in the first 20 centiles and under the introduction/background sections, while 22.22% of the inline citations were found in the last 40 centiles and under the conclusion/discussion sections.

Of the 131,807,433 inline citations that appeared in the main text, 31.06% (40,934,169), 10.36% (13,659,862), 10.89% (14,348,948), and 26.87% (35,419,067) were in the ***I***, ***M***, ***R***, and ***D*** sections, respectively. There were 20.82% of the inline citations (27,445,387 of 131,807,433) that had no IMRaD categories identified. Note that a large portion of citations in the ***NoIMRaD*** category are likely to belong to one of the IMRaD categories because of the lower precision in the ***NoIMRaD*** labelling (see details below). Figure 9 presents the percentages of inline citations in each IMRaD section by text progression. Although the IMRaD sections were identified through a rudimentary approach, the distribution of inline citations was aligned with previous studies^30,48^. Inline citations concentrated at the beginning and the end of scientific articles, and the text progression of IMRaD mainly followed the order of introduction/background, method, result, and conclusion/discussion. In particular, inline citations in the introduction/background mainly appeared in the first 20 centiles, while most of the inline citations identified in conclusion/discussion appeared after the 60^th^ centile. Inline citations identified in method sections showed two lumps, which were around 20^th^-30^th^ centiles and 80^th^-90^th^ centiles. Manual inspection of the submission guidelines of ten journals with high publication counts in the second lump shows that seven journals suggest or require authors to put the method section at the end of the articles. Two journals suggest putting the method section before the conclusion section, but having the conclusion section is not mandatory. One journal has no requirements for the section order.

To gain a deeper understanding of our XML parser’s performance in identifying IMRaD categories, we sampled 100 articles from 100 distinct journals. The IMRaD categories associated with the sentences in the main text of the 100 sampled articles were manually annotated. Precision, recall, and F1 score were computed by comparing the human annotations to the IMRaD categories identified by the XML parser. These indicators were calculated at two different levels: section and sentence. In other words, the performance of the parser was assessed section by section and sentence by sentence. The section-level evaluation was conducted because the IMRaD labels assigned to the sentences were dependent on the section information extracted from the XML files (please see the Method section for details); therefore, if a section was misclassified, all the sentences within it would be misclassified as well.

Table 5 presents the evaluation results. Overall, our parser identified IMRaD categories with high precision but lower recall. The identified ***M***, ***R***, and ***D*** categories were all correct (precision = 1). The precision of the ***I*** category was 99.7% at the sentence level. This was due to four articles with sections titled “Pre-publication history”. The word “history” in the section titles led to their misidentification as ***I*** sections. The macro average recall (93.2%) and the ***NoIMRaD*** precision (48.8%) point to potential improvements: 51.19% of the sentences (1,293 of 2,526) in the ***NoIMRaD*** category belonged to one of the ***I***, ***M***, ***R***, or ***D*** categories. Manual inspection found that the incorrect identification of ***NoIMRaD*** was due to two reasons: (1) the section titles and the XML section information lacked keywords for identifying the IMRaD categories, and (2) ten papers had introduction sections without titles (e.g., PMCID: 4263260). The difference between the sentence level and the section level performances is primarily because the true ***NoIMRaD*** sections tend to be shorter than the IMRaD sections.

**Table 5:** Precision, recall, and F1 of the evaluation results.

Evaluation Level	IMRaD	Precision	Recall	F1
Section	I	0.957	0.854	0.903
	M	1.000	0.820	0.901
	R	1.000	0.915	0.956
	D	1.000	0.993	0.996
	NoIMRaD	0.638	0.949	0.763
	Macro average	0.919	0.906	0.904
Sentence	I	0.997	0.903	0.948
	M	1.000	0.875	0.933
	R	1.000	0.892	0.943
	D	1.000	0.996	0.998
	NoIMRaD	0.488	0.995	0.655
	Macro average	0.897	0.932	0.895

### Alignment between OpCitance and S2ORC

**Fig. 10:**
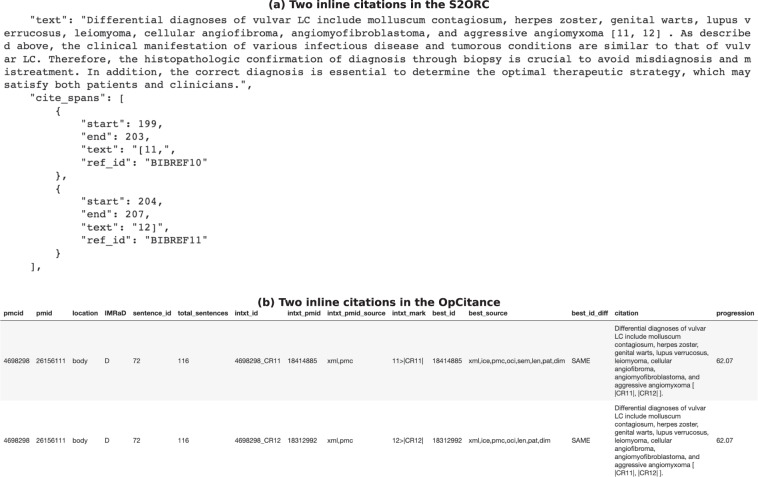
Example of two inline citations in S2ORC and OpCitance.

To our best knowledge, S2ORC is the largest full-text dataset with inline citations annotated. OpCitance annotates inline citations at the sentence level with identifiers embedded in the text, while S2ORC provides character start and end of each inline citation in a paragraph (Fig. 10). To compare the coverage of inline citations in S2ORC and our dataset, we started with the S2ORC articles having PMIDs or PMCIDs. The S2ORC data was retrieved from https://github.com/allenai/s2orc. There were 5,415,731 S2ORC articles with PMIDs/PMCIDs and inline citations. However, significant portion (1,122,520 S2ORC articles) were duplicates. For example, S2ORC paper IDs: 215194089 and 9337105 had the same PMID: 25983392 listed in S2ORC. OpCitance has 2,049,871 articles, of which 1,401,788 (68.38%) match one-to-one with S2ORC, while 487,602 (23.79%) are duplicated in S2ORC, and 160,481 (7.83%) are missing in S2ORC. In other words, although the coverage of OpCitance is smaller, the two datasets are complementary since OpCitance contains articles not in S2ORC. Furthermore, articles in OpCitance have been deduplicated.

Of the 1,889,390 articles in both datasets, OpCitance has inline citations for 99.54% of the references (79,631,699 out of 79,998,620 references), while S2ORC has inline citations for 89.35% of the references (83,075,224 out of 92,973,529 unduplicated references). In other words, the percentage of references with inline citations annotated is 10 percent lower in S2ORC. Furthermore, the PMIDs associated with the inline citations are 81.83% (104,012,041 out of 127,111,995 inline citations) versus 71.92% (93,750,386 out of 130,362,008 unduplicated inline citations) in OpCitance and in S2ORC, respectively. The lower rate of inline citation coverage in S2ORC could influence subject-focused studies (e.g., studies on one article, a few articles, or an author’s articles) since some of the citation contexts mentioning the subject could be missing. The lower rate of PMIDs associated with inline citations could influence studies on the PubMed articles since some of the citation contexts could not be found due to the absence of PMIDs. The lower coverage rate of inline citations in S2ORC also reflects the fact that identifying inline citations in PDF files is more challenging than in the XML files.

To further understand the alignment between the two datasets, we randomly sampled 100 citation contexts from 100 different articles in OpCitance. These 100 citing articles were mapped to 145 S2ORC articles. (55 articles were mapped to one S2ORC article each; 45 were mapped to two S2ORC articles each). Each citation contexts had one or more inline citations, resulting in 300 inline citations in total (i.e., three inline citations per citation context on average). Out of the 300 inline citations, 75 (25%) were not in S2ORC, and five were only found in one of the duplicated articles. The absence of inline citations was due to the following reasons: (1) The mapped S2ORC article had empty or incomplete full text (e.g., absence of part of body text). (2) The citation contexts were not recognized in the S2ORC articles. In these cases, the S2ORC articles had the text but failed to identify inline citations in the sentences. As for PMIDs associated with inline citations, out of the 225 inline citations, 144 citations had PMIDs in OpCitance. However, 21 of these PMIDs were not in S2ORC. One inline citation had different PMIDs in the two datasets (PMID: 28222903 in our dataset; PMID: 28340344 in S2ORC). A manual inspection found that the PMID in our dataset was correct. The PMID listed in S2ORC was the erratum of the actual article. This error might be caused by S2ORC linking bibliographies to articles by similarity score computed between their titles^34^.

Text alignment was also examined. The 225 inline citations found in S2ORC corresponded to 74 unique sentences. The text in the two datasets was nearly the same (e.g., minor variations caused by punctuations; see Table 6). However, two sentences were significantly different in the two datasets. The discrepancies were caused by distorted text, either truncated or inserted.

**Table 6:** Examples of differences in text alignment.

### Challenges and limitations

This study aims to construct a large-scale citation context dataset that can benefit future studies on the motivation, importance, and sentiment of citations. Although the JATS tag set provided standard XML vocabularies for parsing the structure of the PMC open access articles, identifying citation contexts from full-text articles is still challenging. Publishers have different ways of using JATS tags for tagging citations. For example, the JAST guideline mentions that a <ref> tag represents an item in a reference list, and each of the cited work under the item should be separately tagged by <element-citation> or <mixed-citation> tags. When a cited work is mentioned in the full-text, the “rid” attribute of the <xref> tag points to the “id” attribute of a <ref> tag in typical cases. However, in the cases with multiple cited work nested under a <ref> tag, the “rid” attribute could point to the “id” of the <ref> and the “id” of the <element-citation> or <mixed-citation> interchangeably. Another challenge is identifying the implicitly mentioned citations. As described in the method section, the implicitly mentioned citations were inferred from the citation markers containing a hyphen (e.g., [3–6]). However, publishers expressed “hyphen” differently. The “hyphen” could be a hyphen, an en dash (Unicode character U + 2013), a minus sign (Unicode character U + 2212), or two hyphens/en dashes/minus signs.

Using XML tags has limitations. We manually inspected the references without citation context and found the following reasons. First, some citation markers in the full-text articles were plain strings (i.e., these citation markers did not associate with any <xref> tag). In these cases, our XML parser could not pick up the citation contexts. Second, there were cases where the citation marker in the full-text article pointed to a nested reference, but the <xref> tag only pointed to one cited work in the nested reference. Third, some references were not mentioned by the authors in the full-text. However, these conditions were rare. Of all the 86,473,346 references in OpCitance, only 0.51% (437,471) of the references’ citation contexts could not be identified by our XML parser. It is also worth noting that the first two limitations may be improved by developing a text-mining model that can identify citations in sentences and link them back to their references. The annotated citations (i.e., the citation contexts) and their references provided in OpCitance can be used as training data for developing the model. We plan to work on this in our future studies. Another limitation was found in identifying PMIDs of citation by XML tags. In this study, the <pub-id> tag with *pmid* attribute value (*//pub-id[@pub-id-type = ‘pmid’]*) was used for finding the PMIDs of citations in the XML files. Like the citation markers, we discovered that some PMIDs could be found in the citation strings but were not tagged.

Compared to S2ORC, which used machine-learning libraries (Science Parse and GROBID) to parse PDF versions of articles and identify inline citations, our effort focused on developing an XML parser that can handle the nuances of the use of JATS tags by different publishers and identify citation contexts as completely as possible. Our work contributes to parsing scientific papers and identifying inline citations by making the dataset and the XML parser publicly available. The dataset provides 137,748,787 inline citations and their citation contexts, covering 99.49% (86,035,875) of the total 86,473,346 references. The release of the parser enables users to create their own datasets for JATS-standard XML versions of articles. Although the parser is limited to XML documents conforming to JATS and cannot be applied to PDF versions of articles, the parser still has the potential to be used for extracting inline citations and their citation contexts from articles deposited into PMC and published by journals that adopt JATS such as PLOS ONE (https://plos.org/text-and-data-mining/) in the future. The pipeline deals with issues specific to the JATS-standard XML documents and has the capacity to handle the different ways inline citations may be tagged by the publishers. The add-on with Patci has enhanced the completeness of source ID links between the citing and cited articles. As mentioned in the Technical Validation section, 8.8% of inline citations’ source link IDs have been supplemented or corrected by Patci (i.e., the inserted IDs and the swapped IDs). Furthermore, in contrast to S2ORC providing inline citations at the paragraph level, OpCitance provides inline citations at the sentence level. Different annotation levels between S2ORC and OpCitance provide users flexibility to select the dataset that best suits their needs.

## Usage Notes

### Generating features from the dataset

Since Garfield^49^ published fifteen possible citation motives, abundant efforts have been put in developing features for modelling citation motives using citation contexts. For example, Valenzuela *et al*.^44^ and Zhu *et al*.^14^ used 12 and 38 features to model the importance of cited references to the citing articles, respectively. Teufel *et al*.^8^ developed a set of features focusing on semantical similarity and used these features to model citation functions. In the meta-analysis conducted by Kunnath *et al*.^25^, features used in studies on citation function and importance were categorized as noncontextual features (e.g., positional-based and frequency-based features) and contextual features (e.g., syntactic and semantic features). Here, we use Kunnath *et al*.‘s^25^ categories and give examples of codes for generating positional-based and frequency-based features, as well as instructions for generating contextual features. The codes were written in Python3, using pandas library for data processing.

#### Positional-based and frequency-based features

Many of the features related to frequency and location can be obtained from OpCitance dataset through grouping or filtering data. For instance, the number of mentions (i.e., the citation counts in the entire paper) can be acquired from grouping the data by the *pmcid* and *intxt_id* columns:


Number_of_mentions = df.groupby(['pmcid','intxt_id'])[['intxt_id']].count()


For the articles following IMRaD structure, the number of mentions in each IMRaD section^14,44^ can be acquired from grouping the data by the *pmcid*, *IMRaD* and *intxt_id* columns:


Number_of_mentions_by_IMRaD = df.groupby(['pmcid','IMRaD','intxt_id'])[['intxt_id']].count()


Similarly, the number of different IMRaD sections in which citation contexts of a reference were identified can be obtained from:


Number_of_mentioning_IMRaD_sections = df.groupby(['intxt_id'])[['IMRaD']].nunique()


Citations in tables and figure captions^44^ can be obtained from:


Citation_context_in_figure_or_table = 1 – df['location'].isin(['abstract','body','back'])


As for features related to text progression of citation contexts^14^, these features can be calculated through the *progression* column. This column provides the centiles of citing sentences within the main text of articles.

#### Contextual features

Contextual features can be obtained from processing the citation contexts through natural language processing toolkits such as NTLK or Stanford NLP. For instance, the function verbs used in Teufel *et al*.^8^ can be identified from conducting part-of-speech (POS) tagging on the citation contexts. For calculating text-similarity features^14,44^ such as the text-similarity between each citation context and the abstract of the citing article, the abstract of each citing article can be retrieved by selecting the rows with the “abstract” label in the *location* column.

### Other possible applications

With the semantically enriched citations, OpCitance opens up a wide variety of applications. In addition to studying citation motives, functions, and importance, OpCitance can be used for identifying trends in research fields, visualizing scientific landscapes, and analyzing the domain of journals. Many studies on these topics relied on citation links and/or text in article titles and abstracts. For example, Chen and Song^50^ proposed a method for visualizing a scientific field and identifying topic advancement in the field using citation expansion (i.e., tracing forward or backward citations of given seed articles). Wang *et al*.^51^ applied NLP techniques to titles and abstracts to identify emerging topics in nano-publications. Glanzel *et al*.^52^ classified the fields of articles published in multidisciplinary and general journals by mapping journal information indicated in the references to their subject domains. Zhang *et al*.^53^ utilized citations between journals to cluster scientific papers into seven domains. The UCSD map of science^54^ constructed visualizations for scientific fields by clustering journal-to-journal citations and keywords. Waltman and van Eck^55^ proposed a system for identifying research areas based on citations between articles. In their study, article titles and abstracts were used to label the identified research areas^55^. Compared to titles and keywords, citation contexts contain information that is more directly related to the citations. Therefore, analyses that combine citations and citation contexts may yield further insights into detecting and visualizing research trends and domains.

## Data Availability

The code of our XML parser is provided in the Supplementary_File_1.zip on our data repository: 10.13012/B2IDB-4353270_V2.
